# Effect of hepato-toxins in the acceleration of hepatic fibrosis in hepatitis B mice

**DOI:** 10.1371/journal.pone.0232619

**Published:** 2020-05-19

**Authors:** Suchithra Poilil Surendran, Reju George Thomas, Myeong Ju Moon, Rayoung Park, Doo Hyun Kim, Kyun Hwan Kim, Yong Yeon Jeong

**Affiliations:** 1 Department of Biomedical Sciences, Biomolecular Theranostics (BiT) Lab, Chonnam National University Medical School, Hwasun, Korea; 2 Department of Radiology, Biomolecular Theranostics (BiT) Lab, Chonnam National University Medical School, Hwasun, Korea; 3 Department of Pharmacology and Center for Cancer Research and Diagnostic Medicine, Konkuk University School of Medicine, Seoul, South Korea; Harvard Medical School, UNITED STATES

## Abstract

Chronic liver diseases such as hepatitis B viral (HBV) infection and liver fibrosis have been a major health problem worldwide. However, less research has been conducted owing to the lack of animal models. The key purpose of this study was to determine the effects of different hepatotoxins in HBV-affected liver. In this study, we successfully generated a combined liver fibrosis model by administering HBV 1.2 plasmid and thioacetamide/ethanol (TAA/EtOH). To our knowledge, this is the first study in which an increase in the liver fibrosis level is observed by the intraperitoneal administration of TAA and EtOH in drinking water after the hydrodynamic transfection of the HBV 1.2 plasmid in C3H/HeN mice. The HBV+TAA/EtOH group exhibited higher level of hepatic fibrosis than that of the control groups. The hepatic stellate cell activation in the TAA- and EtOH-administered groups was demonstrated by the elevation in the level of fibrotic markers. In addition, high levels of collagen content and histopathological results were also used to confirm the prominent fibrotic levels. We established a novel HBV mice model by hydrodynamic injection-based HBV transfection in C3H/HeN mice. C3H/HeN mice were reported to have a higher HBV persistence level than that of the C57BL/6 mouse model. All the results showed an increased fibrosis level in the HBV mice treated with TAA and EtOH; hence, this model would be useful to understand the effect of hepatotoxins on the high risk of fibrosis after HBV infection. The acceleration of liver fibrosis can occur with prolonged administration as well as the high dosage of hepatotoxins in mice.

## Introduction

All chronic liver injuries are characterized by the presence of liver fibrosis. Hepatic fibrosis results from the inflammation of liver cells as a result of the excessive accumulation of extracellular matrix (ECM) and scarring of the liver tissue. Fibrosis is reversible, while cirrhosis, the advanced form of fibrosis is proven to be irreversible [1–4]. Hepatic stellate cells (HSCs) play a major role in the production of ECM. The increased production of ECM results in the overexpression of fibrotic markers or ECM proteins, such as α-smooth muscle actin (α-SMA), collagen, tissue inhibitors of metalloproteinases (TIMP), etc. The main reason for this is the activation of HSCs from the quiescent form to the myofibroblast-like form during hepatic injuries [5–8].

Chronic infection of hepatitis viruses can also lead to severe hepatic fibrosis and ultimately cirrhosis and cancer [9]. Hepatitis B (HBV) and hepatitis C viral (HCV) infections are considered to be major roots of chronic liver diseases globally. As HBV is a highly necro-inflammatory disease, the possibility of hepatocellular carcinoma (HCC) is relatively high [10–12]. HBV infection results in inflammatory changes followed by the release of different cytokines as well as chemokines such as interleukin-1 and -8 (IL-1, IL-8), interferon-γ, and tumor necrosis factor alpha (TNF-α). These cytokines and chemokines will kill HBV-associated CD8+ cytotoxic T cells. This type of hepatic oxidative stress leads to the activation of Kupffer cells followed by the activation of HSCs results in fibrosis via triggering of different genes [13–15].

The induction of hepatic fibrosis is not easy in mice. Animal models of hepatic fibrosis can be categorized by the etiologic factors, including toxin, nutritional, immunologic, biliary, alcoholic, and genetic factors. The four major types of HBV mouse developed thus far are the HBV transgenic mouse, human liver chimeric mouse, transduction of HBV replicons using adeno-associated virus and hydrodynamic transduction of HBV replicons [16]. These are the widely studied HBV humanized mouse models. However, humanized mouse models are not suitable for understanding the mechanism of HBV viral actions. Inadequate information regarding the mechanism of action of HBV virus limits all the current mouse models [17].

A thioacetamide (TAA)-treated mouse model is related to more noticeable regenerative nodules, which results in the rapid formation of periportal fibrosis leading to a cirrhosis (Schema 1). The main drawback of this model is the time consumption as well as the development of cholangiocarcinoma after 18 weeks of TAA administration. Prolonged consumption of ethanol (EtOH) may results in the advanced hepatic impairment such as simple steatosis, progressive fibrosis, and cirrhosis. TAA and EtOH application was suitable for inducing liver fibrosis in C3H/HeN mice [18]. Both TAA and ethanol act as hepatotoxins, and the formation of liver fibrosis is fast compared to the effect of TAA or ethanol alone. Repeated intraperitoneal injections of TAA and chronic supplementation of EtOH in drinking water cause severe fibrosis and cirrhosis between 16 and 24 weeks in mice. Although animal models of hepatic fibrosis are suitable for studying hepatic fibrosis and cirrhosis regardless of the etiology of fibrosis, this model does not reflect hepatic fibrosis caused by hepatitis, the most common cause in clinical practice. There is a high influence of alcohol and hepatotoxins on the elevation of liver fibrosis in HBV-infected patients [19].

**Schema 1:**
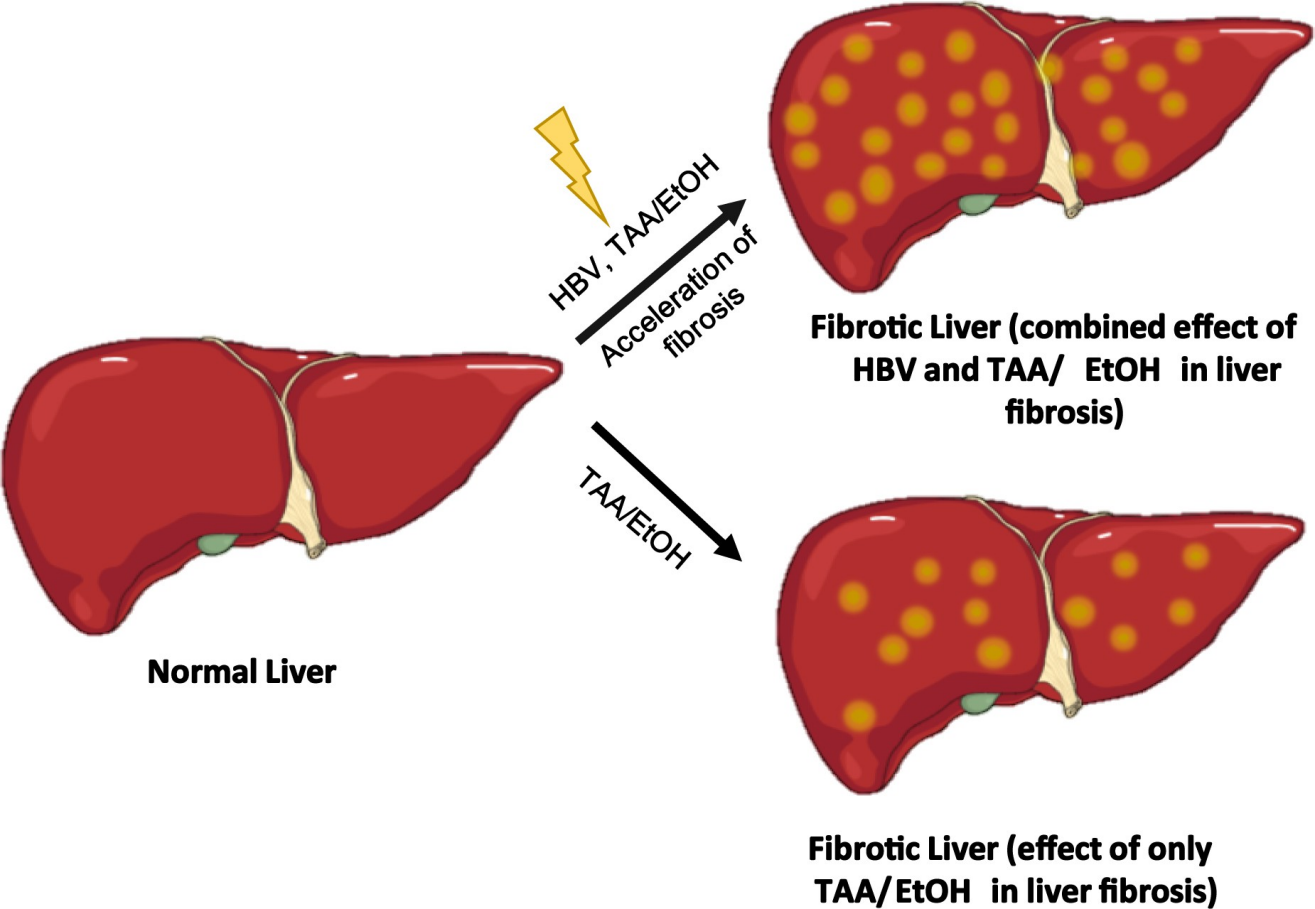
Acceleration of hepatic fibrosis in HBV-TAA/EtOH-treated mice.

To our knowledge, there is no animal model reported to date that can be used to study all the mechanisms involved in hepatic fibrosis as well as the interplay between hepatotoxins and HBV [20]. Therefore, we hypothesized that this combination of HBV and hepatotoxins in the acceleration of hepatic fibrosis could be useful for an improved understanding of the interrelation between HBV and hepatotoxins. The goal of this study was to establish a mouse model by the hydrodynamic injection (HI) of the HBV 1.2 plasmid after the administration of TAA and EtOH to evaluate the combined effect of EtOH and other hepatotoxins in the acceleration of liver fibrosis in HBV mice.

## Materials and methods

### Materials

Thioacetamide was purchased from Sigma–Aldrich, USA. DAB substrate and DAB staining solution were purchased from BD Pharmingen (San Diego, CA 92121, USA). The HepG2 and FL83B cell lines were purchased from ATCC (Manassas, USA). Dulbecco’s modified Eagle’s medium (DMEM) was purchased from Thermo Scientific, USA. L-Glutamine (200 mM) was purchased from Sigma–Aldrich, USA. A hydroxyproline assay kit was purchased from Chondrex (WA, USA). Anti-alpha smooth muscle actin antibody-Lot:GR283004-24 (Abcam, Cambridge, UK), goat anti-rabbit IgG H&L (Alexa Fluor1 488) Lot:GR306624-1 (Abcam, Cambridge, UK), hepatitis B core antigen Lot:#SF2406841H (Invitrogen, Thermo Fisher Scientific, South Korea), and goat anti-rabbit IgG (HRP) Lot:GR247075-7 (Abcam, Cambridge, UK) were used. A plasmid DNA purification kit was purchased from Intron Biotechnology (Lynnwood WA). The DNA isolation kit was purchased from QIAamp (DNA Mini kit, Germany). An ELISA kit for HBV antigen analysis was purchased from Wanti-Biopharm (Beijing). All other reagents used were of analytical or chromatographic grade.

## Methods

### In vitro studies

#### Expression of plasmid in vitro

FL83B cells were seeded in six-well plates at 4x10^5^ cells per well with high-glucose DMEM containing 10 vol% FBS, penicillin-streptavidin-amphotericin B (Gibco Antibiotic-Antimycotic (100X), USA) as the culture medium. Using Lipofectamine 2000 (Invitrogen, Carlsbad, CA, USA), the replication-competent HBV 1.2mer (HBV 1.2) plasmid [21,22] was transfected into the FL83B cell line according to the manufacturer’s protocol. After transfection, the supernatant of the transfected cells was collected every day for five days, and the HBV markers HBsAg and HBeAg were determined by ELISA using an ELISA kit. The supernatant was replaced by the same amount of media.

#### DNA isolation of HBV plasmid

The HBV 1.2 plasmid was transfected into the FL83B cell line using the manufacturer’s protocol, and the transfection efficiency of the plasmid was checked. The transfected cells were harvested after three days, and nucleic acid was extracted using a DNA isolation kit (QIANGEN). HBV viral DNA was quantified using qPCR. All qPCR analyses were performed on a QuantStudio3, Applied Biosystems, (Thermo Fisher Scientific, Waltham, MA, USA). The analysis of the qRT-PCR results was performed on the basis of a dissociation replication plot.

## In vivo studies

### Mice

All our studies were performed in 5 week old C3H/HeN female mice (n = 115, 18 to 20 g body weight) purchased from Jungang Lab Animal, Inc., Korea. The Chonnam National University Medical School Research Institutional Animal Care and Use Committee approved the experimental protocol (CNUHH 2014–148). All the mice were housed in plastic cages in a semi specific pathogen free facility under controlled conditions 22^0^c temperature and 12 hour light-dark cycle. All the experiments were carried out by researchers trained for animal handling according to the Chonnam National University Medical School Research Institutional Animal Care and Use Committee protocol. The behavioral changes of the mice were monitored twice in a day, which includes the weight loss measurements, fur loss, hunching and immobilization checking etc. The weight of the mice were maintained to a minimum of 20 grams, mice below that weight were handled and monitored carefully until it shows a dramatic loss in the weight. In some mice this dramatic change in the weight up to 15 g with difficulties in mobilization and fur loss were observed. These mice were sacrificed before finishing the experiment schedule. Other mice which maintained their weight were sacrificed at the end of the experimental schedule. CO_2_ was used as the euthanasia agent for sacrificing all the mice. After the CO_2_ asphyxiation the euthanasia was confirmed by the physical examination of heartbeat and respiration.

### HBV+TAA/EtOH mouse model of hepatic fibrosis

The mice were divided into four different groups: control (n = 24), TAA/EtOH (n = 27), HBV (n = 24) and HBV+TAA/EtOH (n = 40). The HBV and HBV+TAA/EtOH mouse groups were hydrodynamically injected with HBV 1.2 plasmid [21,22]. In detail 25 μg of HBV1.2 was injected via tail vein in saline of volume of 10% body weight of the mouse. Intraperitoneal administration of TAA and oral delivery of EtOH in drinking water were started one week after transduction of the plasmid into the liver of the HBV+TAA/EtOH group and the TAA/EtOH group. The weighed TAA was solubilized in saline and used for intraperitoneal injections. The mice were given anesthetics before injections and all mice health were monitored daily. The control group represents the mouse group without any treatment. All hepatic fibrosis model groups were created by the weight-adapted administration of TAA and EtOH. The doses of TAA and EtOH were determined after checking the weight of each mouse every week (**S1 Fig in S1 File**). At the same time, 10% ethanol in drinking water was given to the mice. All mice were sacrificed at various time points according to the study, the liver was harvested, and blood was collected using the cardiac puncture method. Our study exhibited a 10% mortality in the HBV+TAA/EtOH mouse group and 8% mortality in both the TAA/EtOH and HBV mouse groups. The main humane endpoint for euthnasia we considered was weight loss in mice after TAA/EtOH administration, as weight loss is a reported visible effect in mice after hepatotoxin administration [18]. Therefore TAA and EtOH was injected in a controlled manner with careful monitoring of mice health and weight. Even though weight loss was considered as the specific criteria (humane endpoint) to determine when animals should be euthanized, 6 mice were dead from a total of 115 mice for the duration of 15 weeks. The cause of death of mice might be the over sensitiveness to the hepatotoxins. We have conducted two different sets of studies that provide an understanding of the effect of time duration on hepatotoxin administration as well as the effect of dosage on hepatotoxin administration.

#### Time-dependent study

Time-dependent groups of mice were created to assess the effect of TAA and EtOH on the progression of liver fibrosis at different time points. The same four mouse groups as above were created. After transduction of the plasmid, TAA and EtOH administration started, and mice were sacrificed at different time points, such as 1, 3, 6, 9, 12 and 15 weeks. The difference in progression of liver fibrosis was analyzed using blood and liver from each group.

#### Dose-dependent study

Dose-dependent groups of mice were created to check the effect of TAA dosage. TAA was administered at different dose levels, and the mouse model HBV+TAA/EtOH was again divided into low dosages (80 to 210 mg/kg of TAA), medium dosages (90 to 300 mg/kg of TAA), and high dosages (100 to 400 mg/kg of TAA). In addition, 10% EtOH was given in drinking water. All mice were sacrificed after 12 weeks of TAA and ETOH administration, and blood was collected for ELISA, Blood biochemical analysis and liver for qPCR and other histopathological studies.

Schema 2 represents the in vivo studies performed in this work. Hydrodynamic injection of HBV 1.2 plasmid was performed followed by TAA and EtOH administration after 1 week. The administration of TAA and EtOH was performed based on the weight-adapted method [23]. For the time-dependent study, the mice were sacrificed at different time points, including 1, 3, 6, 9, 12 and 15 weeks, for further studies. At the same time, mice were administered different doses of TAA and EtOH and sacrificed at 12 weeks for the dose-dependent studies.

**Schema 2:**
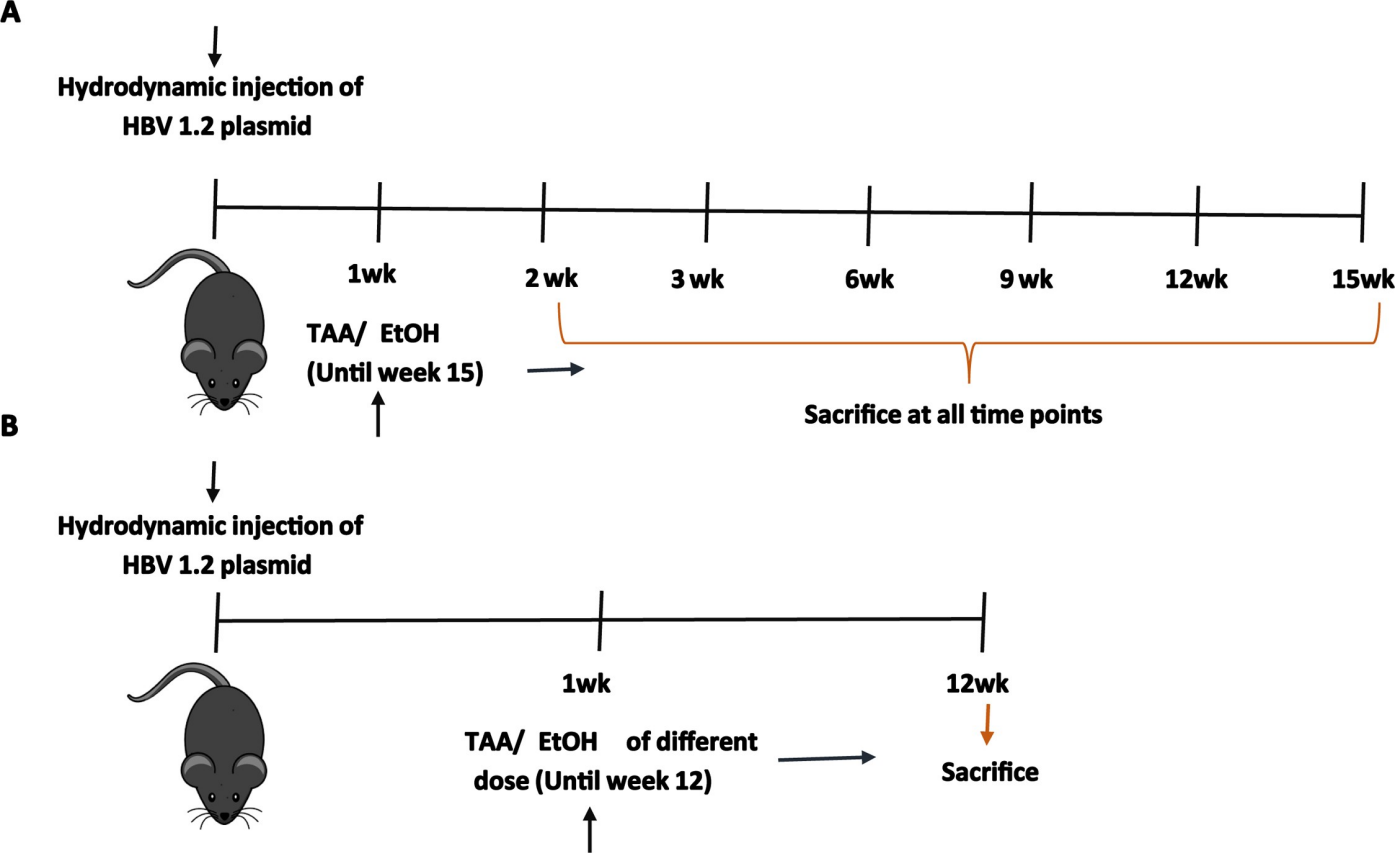
In vivo study schema of (A) the time-dependent TAA/EtOH administration in HBV-infected mice and (B) the dose-dependent TAA/EtOH administration in HBV-infected mice.

### Expression of HBsAg and HBeAg

The HBV markers HBsAg and HBeAg were determined using ELISA. The blood serum was used for the determination, and commercially available kits were used as per the manufacturer’s instructions. The ELISA test was done considering negative as well as positive controls provided in the kit. The colorimetric analysis and quantification method was used to determine the presence of HBeAg and HBsAg. Negative control, positive control, blank and the samples were treated with HRP-conjugate and incubated for 60 minutes followed by washing. To generate the color the provided chromogen solutions were added and the incubated for 30 minutes. The reaction was stopped by adding stopping solution and the absorbance was measured using a plate reader at 450nm wavelength. The specimens with absorbance to cut off value higher than 1.1 is considered to be positive to HBsAg and HBeAg.

### Quantification of HBV DNA

All mice were sacrificed, and harvested liver samples were used to determine HBV DNA levels. Twenty-five micrograms of liver samples was made into powder using liquid nitrogen followed by DNA isolation using a QIAamp DNA Mini kit (Qiagen, Germany). The resulting DNA samples were stored at -80°C and used for the qPCR experiments [24]. All qPCR reactions were performed in triplicate in 96-well hard shell PCR plates using SYBR Green PCR master mix from Applied Biosystems (Thermo Fisher Scientific, Warrington, UK). The primers used for the study are given in **S1 Table in S1 File**.

### Blood biochemistry

Mice were sacrificed, and the blood was collected by the cardiac puncture method. One milliliter of collected blood was centrifuged at 3500 RPM for 15 minutes, and the blood serum was collected. Blood serum samples were used to check the blood biochemistry levels of ALT, AST, LDH and CK.

### Histopathological analysis

Mouse livers were harvested at different time points and fixed in 10% formalin. Using standard tissue processing techniques, all the tissue samples were embedded in paraffin. Histopathological studies related to fibrosis markers and HBV markers were performed in paraffin-embedded 6-μm-thick tissues. HBV core antigen expression in the hepatic tissue was detected immunofluorescence staining. Hematoxylin-eosin (HE) and Masson trichrome staining were performed to confirm the level of fibrosis in the histological samples. At the same time, an immunofluorescence assay was used to check the expression of α-SMA.

### Hydroxyproline content

The collagen content in the liver was determined using the indirect evaluation of hydroxyproline content using the hydroxyproline kit. Collagen levels in the liver of different groups were checked. Ten milligrams of liver samples from all the groups was smashed with 100 μl of distilled water to obtain a paste. The samples were hydrolyzed for 24 hours with 100 μl of 10 N HCl. Then, all the sample supernatant was collected after centrifugation at 10,000 rpm for 5 minutes. One hundred microliters of 1X chloramine T solution was added to 10 μl of the supernatant and incubated at room temperature for 20 minutes. After this step, 100 μl of 1X DMAB solution was added and incubated for 30 minutes, and the optical density at 530 nm was measured using a microplate reader. The results were used to calculate the hydroxyproline content in the liver [25].

### Western blotting

Western blotting analysis of alpha-SMA was performed to confirm the fibrosis level in the mice. The mice were sacrificed, and the liver was collected for further experiments. The liver lysates were separated using 12% SDS-polyacrylamide gels followed by transfer to a polyvinylidene fluoride membrane (PVDF) (Immobilon-P, USA) as per the standard procedures. After transferring the membranes, the membranes were blocked using 5% normal horse serum in PBS containing 0.02% sodium azide for 1.5 hours. Primary antibodies diluted in blocking solution were added and incubated overnight at 4°C. The blotted membranes were washed 3 times with TBST for 10 minutes each time and incubated with secondary antibody diluted in TBST for 1.5 hours at room temperature. After incubation, the membrane was again washed with TBST. Protein bands were detected using western blotting luminol reagent (Santa Cruz Biotechnology, USA).

### Statistical analysis

Statistical analysis was established using GraphPad Prism 5.03 software (GraphPad Software 2365 Northside Dr. Suite 560 San Diego, CA 92108). The data indicated as the mean ±SD of 5 independent experiments per each group. The *** represents a p value < 0.005, ** indicates p <0.01, * shows p < 0.05, and p > 0.05 is considered to be nonsignificant. The term ns represents non–significance. The Alpha was set to 0.5 and the comparison of two groups was performed by student’s t-test at the same time comparison of data sets were done by a 2-way ANOVA test.

## Results

### Expression of HBsAg and HBeAg

**Fig 1:**
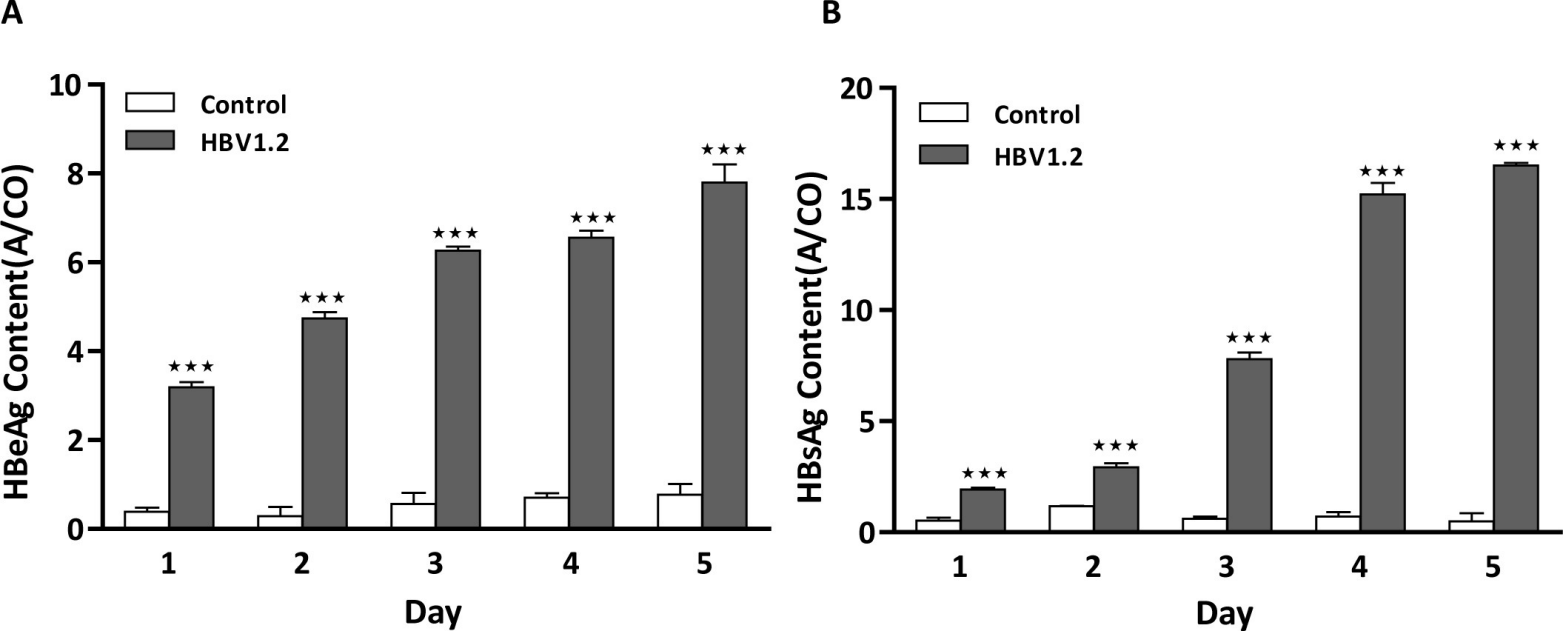
(A) HBeAg and (B) HBsAg contents in the supernatant of HBV 1.2-transfected cells from day 1 to day 5 compared with non-transfected cells. The antigen contents were increased according to days indicates the successful transfection of the plasmid in vitro.

Transfection of the HBV 1.2 plasmid in the FL83B cell line was determined by ELISA assay of HBeAg and HBsAg [26]. The presence of both HBeAg and HBsAg markers in the supernatant of the transfected cell media was used to confirm the transfection of the plasmid in hepatocytes. After transfection, from day 1 to day 5, HBeAg **(Fig 1A)** and HBsAg **(Fig 1B)** levels were increased every day compared to levels in the non-transfected cells. After day 1, the levels of both HBV markers increased until day 5 compared to the levels of the non-transfected one. As the expression of surface antigens are generally higher compared to HBeAg, the A/CO values of HBsAg is slightly higher than that of HBeAg. This result exhibits the effective transfection of the HBV plasmid in FL83B cells and can be used for the further studies in vivo.

### Quantification of HBV DNA

**Fig 2:**
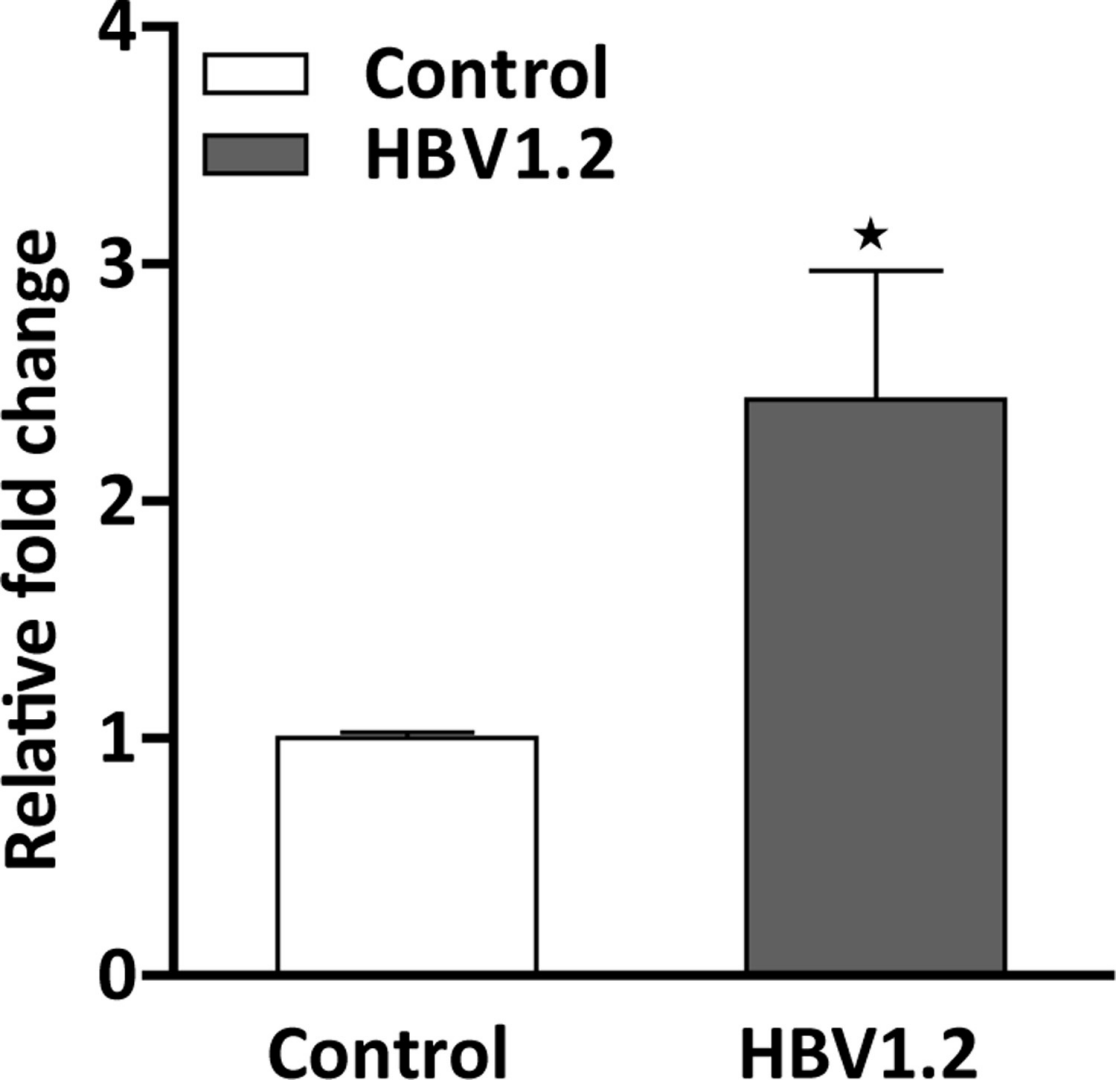
qPCR quantification of the HBV 1.2 DNA content in transfected cells compared to that in the non-transfected cells. The quantification showed a 2.4 fold increase in the HBV DNA in the plasmid incubated cells reveals the successful transfection in vitro.

The HBV 1.2-transfected FL83B cell lines were used to quantitatively analyze the transfection by determining the HBV DNA present in the cell line [27]. The transfection of the HBV 1.2 plasmid in the FL83B cell line was quantified using qPCR **(Fig 2).** The plasmid was successfully transfected into the cells when compared with that of the control group. These results show the efficiency of the transfection of the plasmid into hepatocytes and the successful replication in hepatocytes. The qPCR result exhibited a 2.4-fold increase in HBV plasmid expression when compared to expression in the non-transfected cells. This results can be considered for the in vivo studies of plasmid transduction in mice hepatocytes.

## In vivo studies

### Expression of HBsAg and HBeAg

**Fig 3:**
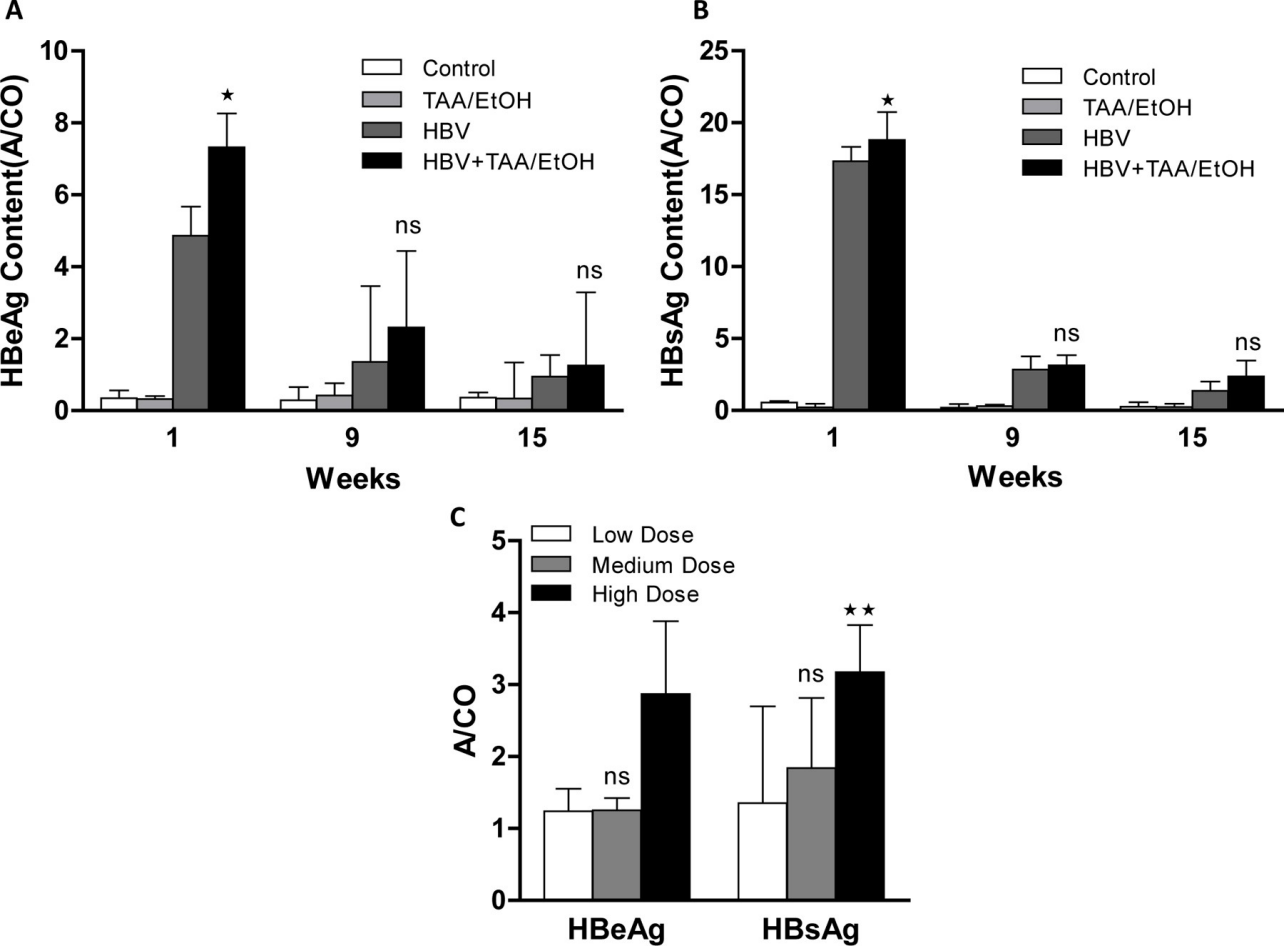
(A) HBeAg and (B) HBsAg levels in the blood at different time points. (C) HBeAg and HBsAg levels in the blood with different dosages of TAA/EtOH. The HBsAg and HBeAg quantification in the combined mice group showed a slight increased expression.

The presence of HBsAg and HBeAg in blood are the major indicators of HBV infection in the liver. ELISA was used to check the presence of HBsAg and HBeAg in the blood of mice at different time points of TAA and EtOH administration **(Fig 3A and 3B).** After week 3, both the antigen levels in the blood were reduced in the HBV and HBV+TAA/EtOH groups. This finding represents the elimination of the plasmid after 3 weeks. This elimination is again confirmed by qPCR analysis of HBV DNA.

Both HBeAg and HBsAg were absent in the control and TAA/EtOH groups. However, the ELISA showed the presence of HBeAg and HBsAg in the HBV and HBV+TAA/EtOH groups. These findings confirmed the effective transfection of the plasmid in the hepatocytes of the HBV and HBV+TAA/EtOH groups. The antigen levels were high in the HBV +TAA/EtOH group compared to levels in the HBV group at all time points. This result provides evidence of the increased persistence of HBV DNA with the combined effect of hepatotoxins. From week 1 to week 15, there was a slight increase in the antigen levels in the HBV+TAA/EtOH group compared with levels in the HBV group. ELISA analysis of the dose-dependent mouse groups showed much lower antigen levels in the blood after 12 weeks of TAA and EtOH administration. After 3 weeks of hepatotoxin administration, the HBV plasmid started to be eliminated, and therefore, the antigen levels were also found to be decreased according to time **(Fig 3C).** In contrast, the HBeAg and HBsAg levels in the HBV+TAA/EtOH group showed a slight increase compared to levels in the HBV group, which explains the presence of more HBV DNA in the HBV+TAA/EtOH group than in the HBV group. Overall it is evident that the effect of hepatotoxins in hepatitis B mice will have more adverse effect than the control groups such as hepatotoxins only and HBV only.

### Quantification of HBV DNA

**Fig 4:**
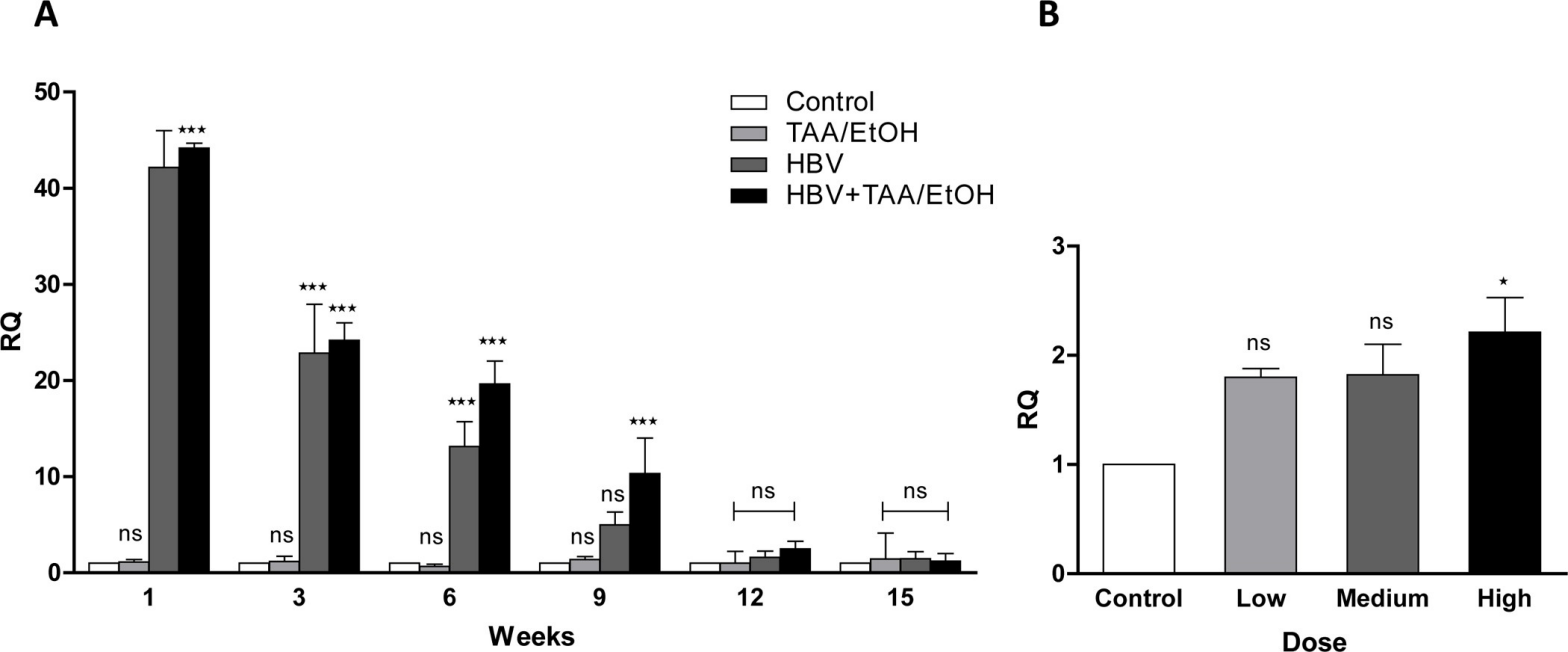
(A) HBV DNA quantification at different time points of TAA/EtOH administration using qPCR analysis. (B) HBV DNA quantification in the dose-dependent study at 12 weeks using qPCR analysis. Both the time depend and dose dependent study exhibited an increased HBV DNA levels in the combined groups compared to the control groups.

HBV DNA was isolated from the liver at different time points, and the levels of HBV DNA were checked. The ELISA showed decreased HBsAg and HBeAg levels after 3 weeks. The qPCR results were similar to the ELISA results proving the elimination of HBV DNA after 3 weeks. The results were in support of all other experimental results, which showed an increased HBV DNA content in the HBV+TAA/EtOH group compared with the content in all the other groups at all time points. Although the level of viral replication is gradually decreased after transduction with the HBV 1.2 plasmid, it persists for at least 9 weeks. In the HBV+TAA/EtOH mouse group, HBV DNA levels were found until week 9. However, in HBV mice, HBV DNA was completely eliminated after week 6 **(Fig 4A).** This shows the persistence of HBV DNA in the combined mouse model compared with that of the other groups. The dose-dependent mouse group revealed that the high-dose group showed a slight increase in HBV DNA levels compared with levels in the low- and medium-dose groups **(Fig 4B).** In contrast, the low dose and medium dose did not show any difference in HBV DNA levels. Eventhough the HBV DNA started eliminating after few days the fibrosis and persistence of HBV DNA in the combined mice model was evident.

### Blood biochemistry

**Fig 5:**
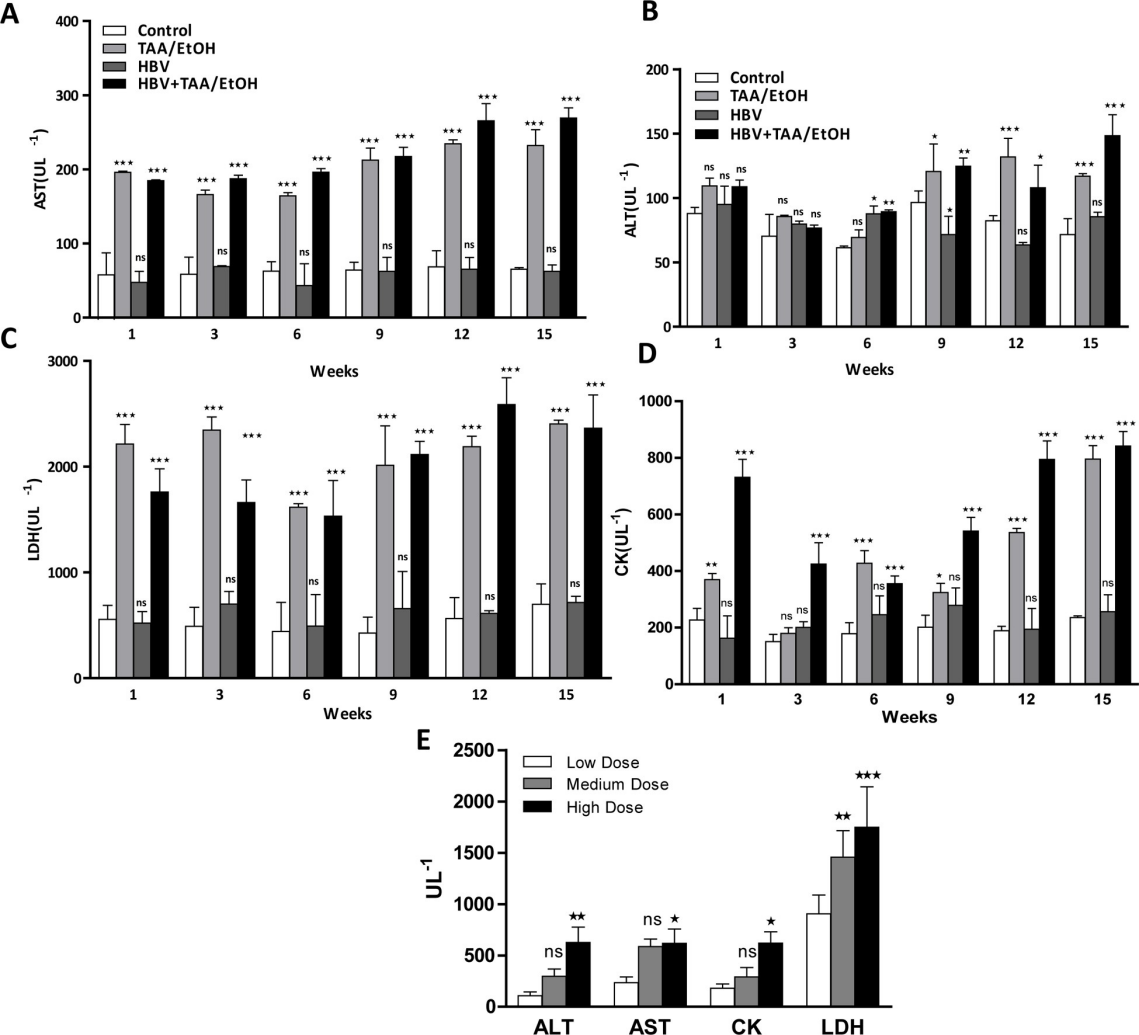
Blood biochemical analysis in the time-dependent study. (A) AST, (B) ALT, (C) LDH and (D) CK. (E) Blood biochemical analyses in the dose-dependent study. The high levels of blood biomarkers in the hepatotoxin treated groups than the control mice exhibit the effect on hepato toxins on mice.

All blood biochemical markers, such as AST, ALT, TK and LDH, were increased in the HBV+TAA/EtOH group compared with markers in the other control groups in the time-dependent study **(Fig 5A, 5B, 5C and 5D).** This finding indicates the connection between hepatotoxins, especially ethanol, and HBV infection in the boosting of fibrosis. The AST/ALT ratio indicates the level of liver fibrosis and was found to be higher in the HBV+TAA/EtOH groups compared to those of the control groups. All blood biochemical markers exhibited an increased level in the high-dose group compared to the low- and medium-dose groups **(Fig 5E).** There was a visible trend in the increasing levels of ALT, AST, CK and LDH from the low dose to the high dose.

### Histopathological analysis

**Fig 6:**
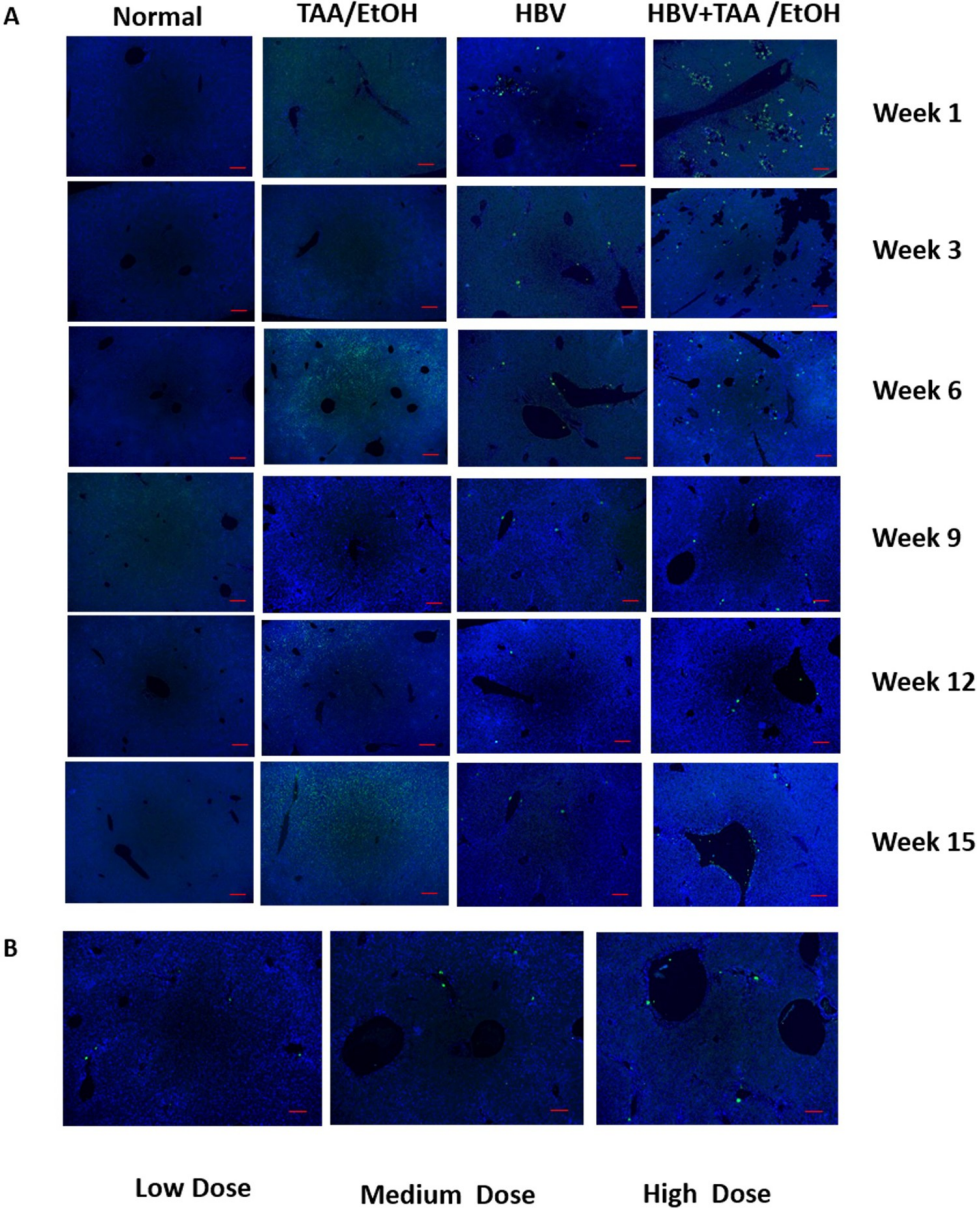
(A) HBV core expression at different time points after TAA/EtOH administration by IHC fluorescence staining. (B) HBV core expression at different dosages of TAA/EtOH administration. The presence of HBV core antigen by IHC can be considered as the reason for increased fibrosis in the combined mice model.

The presence of HBV core antigen within the nuclei was visualized using Immunohistochemical (IHC) staining of the liver sections. The presence of HBV core antigen was found to be maximum at week 3 in the HBV+TAA/EtOH group, and later, it was found to have decreased due to the elimination of HBV plasmid. In the HBV group, the number of HBV core antigens was less than that of the HBV+TAA/EtOH group. The number of HBV core antigen nuclei with green fluorescence was observed to be higher in the HBV+TAA/EtOH group than the number in the HBV group (**Fig 6A**). At all the time points in the study, the presence of HBV core in the HBV+TAA/EtOH group suggests the influence of hepatotoxins in the persistence of HBV DNA as well as the risk of increased liver fibrosis. The spherical core antigen was visualized using IHC staining of liver sections from different dosage groups (**Fig 6B**). The number of HBV core antigens at high doses was higher than those at medium and low doses. The data show that dose-dependent administration of TAA and EtOH in HBV mice resulted in increased liver fibrosis. The dosage also contributed to the replication and persistence of the HBV plasmid.

### Hydroxyproline assay

**Fig 7:**
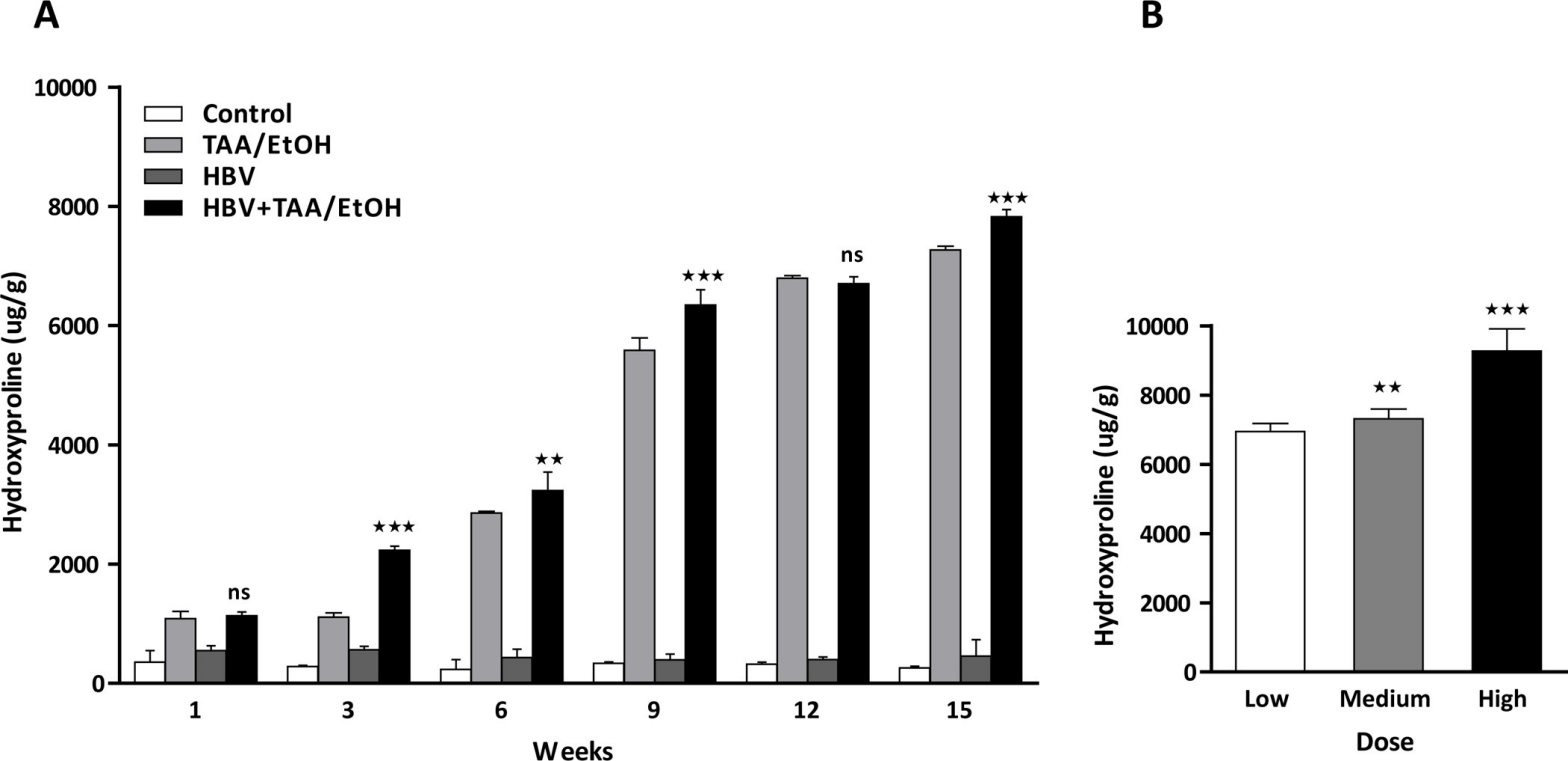
(A) Hydroxyproline content at different time points of TAA/EtOH administration. (B) Hydroxyproline content at different dosages of TAA/EtOH administration. The increased hydorxyproline content in the time and dose dependent study exhibited an indirect measurement of collagen in the liver.

The hydroxyproline assay results showed an increased collagen content in the TAA- and EtOH-treated groups. The HBV+TAA/EtOH group showed more collagen content in the liver compared to the content in the other control groups (**Fig 7A**). Moreover, there was an increase in collagen content with time. This finding indicates that increased fibrosis is associated with increased consumption of hepatotoxins along with HBV infection.

The collagen level in the dose-dependent study also showed a similar result as other collagen detection studies [28]. With increased doses of TAA and EtOH, the collagen level also increased in the HBV+TAA/EtOH group (**Fig 7B**). This suggests that fibrosis will increase with the dosage of hepatotoxins in the combined mouse model of HBV and TAA/EtOH. The high-dose group exhibited a markedly higher amount of bridging fibrosis, which resulted in a high level of collagen content in these groups. The indirect measurement of collagen exhibited a higher fibrotic collagen deposition in the combined group, which suggest the successful animal model generation.

### Western blotting

**Fig 8:**
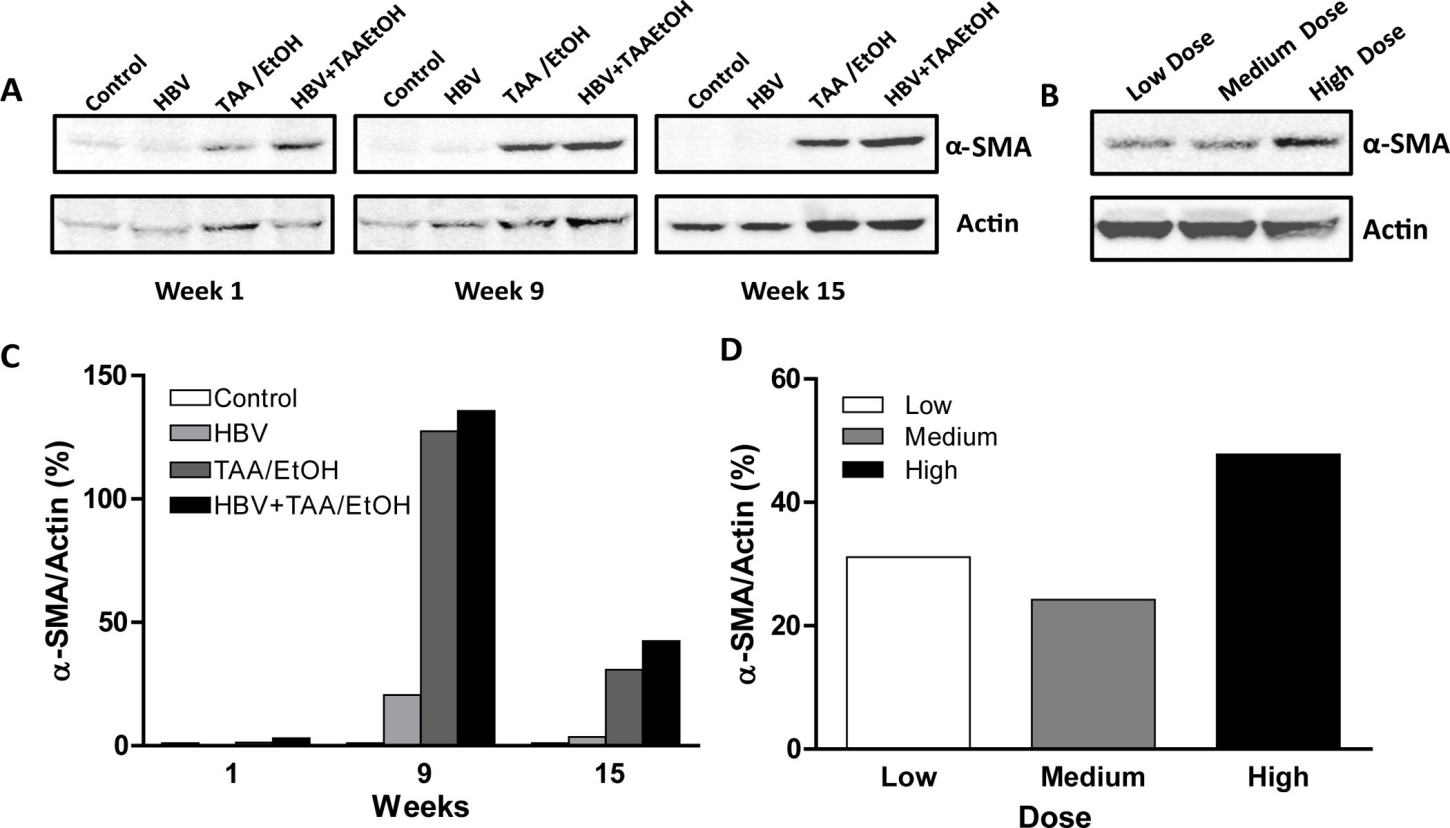
(A) α-SMA expression by western blotting at different time points of TAA/EtOH administration. (B) α-SMA expression by western blotting at different doses of TAA/EtOH. (C) Quantification of α-SMA western blot for time dependent study. (D) Quantification of α-SMA western blot for dose dependent study. The α-SMA analysis by western blotting showed an increased expression in the combined group than the control groups. The difference in expression at different time points and different dosage was evident.

Western blotting analysis in the time-dependent study showed increased alpha smooth muscle actin expression at increased time points. By week 15, α-SMA expression increased as the level of fibrosis increased. Among all the groups, HBV+TAA/EtOH showed increased α-SMA expression compared to the other groups (**Fig 8A**). The dose-dependent study also showed increased α-SMA expression according to the increase in dosage of hepatotoxins. Compared to the low-dose and medium-dose groups, the high-dose-treated group showed more α-SMA expression (**Fig 8B**). The quantitative analysis of western blotting of both time dependent and dose dependent studies were determined (**Fig 8C and 8D).** The -SMA expression was confirmed using IHC staining of the liver (**S3 Fig in S1 File**).

## Discussion

The mouse model was established to prove the effects of the duration and dosage of hepatotoxins on the acceleration of liver fibrosis in HBV-infected mice. We successfully created a mouse model with 10% mortality in the HBV+TAA/EtOH group as well as an 8% mortality rate in the HBV and TAA/EtOH groups. Overall, a greater than 90% success rate was achieved in mouse model creation. The effect of the duration and dosage of hepatotoxins enhanced the acceleration of fibrosis in HBV-infected mice.

The metabolism of alcohol also contributed to TAA metabolism by CYP2E1. Alcohol is first oxidized to aldehydes, and these aldehydes are highly toxic and promote the production of different inflammatory cytokines. At the same time, these aldehydes will activate the transforming growth factors, followed by the synthesis of collagen, which increases the risk of liver fibrosis [29,30]. The major reasons for the elevation in HBV viral replication after alcohol consumption were observed to be due to oxidative stress and hepatic lipid peroxidation, as well as proinflammatory cytokine production followed by a weakening of the immune system. Moreover, alcohol consumption or other hepatotoxin leads to the production of chemokines and cytokines because of the recruitment of macrophages and neutrophils. This production of chemokines and cytokines will result in the inflammatory reaction followed by activation of HSCs and deposition of extracellular matrix. This is also considered to be the main reason for the increase in hepatic fibrosis in HBV mice after hepatotoxin administration [31].

To understand the in vivo transfection efficiency of the HBV 1.2 plasmid in liver cells, we checked the transfection efficiency of the plasmid in HepG2 cell lines and isolated HBV DNA from the cells after transfection and quantified it. The transfection of HBV 1.2 plasmid was confirmed by the detection of HBsAg and HBeAg by ELISA. HBsAg and HBeAg were increased until the fifth day after transfection, which indicated the effective transfection of the HBV 1.2 plasmid into liver cells [32–34].

The blood biochemical results showed increased ALT and AST in the 15-week time-dependent study. The dose-dependent study also showed increased blood biochemical markers in the high-dose-treated groups compared to levels in the other groups. Presence of liver necrosis was assumed by the increased levels of both ALT and AST. The AST and ALT level results in the control group were consistent with other reports. The reason for the increased blood biochemical marker expression is due to the elevation in fibrosis, which results in the loss of hepatocyte membrane integrity [35,36].

In vivo ELISA analysis of HBsAg and HBeAg in both the time- and dose-dependent studies showed a similar trend. After transduction, the plasmid started to be eliminated, but some of the plasmid persisted until week 9. The ELISA results exhibited an increased expression of both antigens in the first week of plasmid injection, after which it started to be reduced over time. In the dose-dependent study, the HBV+TAA/EtOH group also showed more persistent antigen expression due to the increased transduction of the plasmid in the group [37,38].

Quantification of HBV DNA in the liver was carried out using qPCR. The HBV+TAA/EtOH groups showed more persistence of the HBV DNA in the liver for a long time compared to the other study groups. With increased time, the elimination of plasmid was found; however, significant persistence was found in the combined mouse model group. The high-dose-treated group showed more expression of HBV DNA in the liver compared to the low- and medium-dosage-treated groups. A similar trend was observed in HBV core antigen detection. DAB staining of HBV core antigen was carried out to visualize the transfected hepatocytes. HBcAg detected in the HBV+TAA/EtOH group was more prominent in the combined group, but the number of HBcAg was found to be decreased according to time [39–41].

The accumulation of fibrotic tissue in hepatic stellate cells results in α-SMA expression, which indicates the level of fibrosis in the liver. In our study, it was clearly visible that the α-SMA levels were increased according to the duration and dosage of administration of hepatotoxins, which results in the increased accumulation of fibrotic tissues in HSCs. Immunohistochemical staining of α-SMA exhibited similar results as previous studies [42–44].

The H&E results displayed increased inflammatory infiltration into the portal and central areas, and linear bridging fibrosis was observed as the duration of administration of hepatotoxins and dosage of hepatotoxins increased. In the HBV+TAA/EtOH group, bridging fibrosis was more visible by 15 weeks in the time-dependent study. The high-dose-treated group showed increased bridging fibrosis and inflammatory infiltration compared to the low- and medium-dose groups [45–47]. A similar result was found in the western blotting analysis of alpha-SMA, which confirmed the alpha-SMA expression in the HSCs [48–51].

The collagen content in the fibrotic liver by the hydroxyproline assay gives an indirect quantification of collagen in the tissue. In the fibrotic liver, collagen is found to be expressed more due to the activated HSCs, but this will change to highly proliferative quiescent cells, followed by the deposition of collagen type I, which contains high amounts of fibrotic matrixes. This accumulated fibrotic matrix collagen can be quantified using the hydroxyproline assay [52–54]. Our study revealed that the HBV+TAA/EtOH group showed increased accumulation of collagen, while the high-dose-administered mouse group showed high expression.

Studies have shown that the level of HBV markers in HBV-affected patients is extremely high in the case of alcoholic patients compared with markers in chronic liver patients. In our study, we developed a fibrosis combined mouse model with hepatitis B and hepatotoxins. We demonstrated an increased fibrosis rate in HBV mice with the administration of hepatotoxins. The degree of hepatic fibrosis increased with time and dosage of hepatotoxins [55–57]. There is a high influence of alcohol and hepatotoxins on the elevation of liver fibrosis in HBV-infected patients [19]. Both TAA and ethanol act as hepatotoxins, and the formation of liver fibrosis was rapid compared to the effect of TAA and ethanol alone.

## Conclusion

We established a novel HBV hydrodynamic injection-based C3H/HeN mice model with higher HBV persistence level than C57BL/6 mouse model. Our study model can be utilized to understand the effect of alcohol in HBV mice, which is useful for better understanding the risk of drinking alcohol in HBV-positive patients and their liver fibrosis progression. The acceleration of liver fibrosis can occur with prolonged administration as well as a high dosage of hepatotoxins in mice.

## Supporting information

S1 File (DOCX)
