# Supplementary material for: Effect of hepato-toxins in the acceleration of hepatic fibrosis in hepatitis B mice
Source: PLoS One. 2020 May 19;15(5):e0232619. doi: 10.1371/journal.pone.0232619 (PMC7237019; doi:10.1371/journal.pone.0232619)
Supplement: S1 File — (DOCX) [file pone.0232619.s001.docx]

***Supplementary Information***

***Effect of Hepato-Toxins in the Acceleration of Hepatic Fibrosis in Hepatitis B Mice***

Suchithra Poilil Surendran ^1,2^, Reju George Thomas ^1,2^ , Myeong Ju Moon ^2^ , Rayoung Park ^2,^ Doo Hyun Kim^3^, Kyun Hwan Kim ^3^ and Yong Yeon Jeong ^2,*^

***Weight adapted TAA administration***

The dosage of TAA was decided based on the weight adapted method.


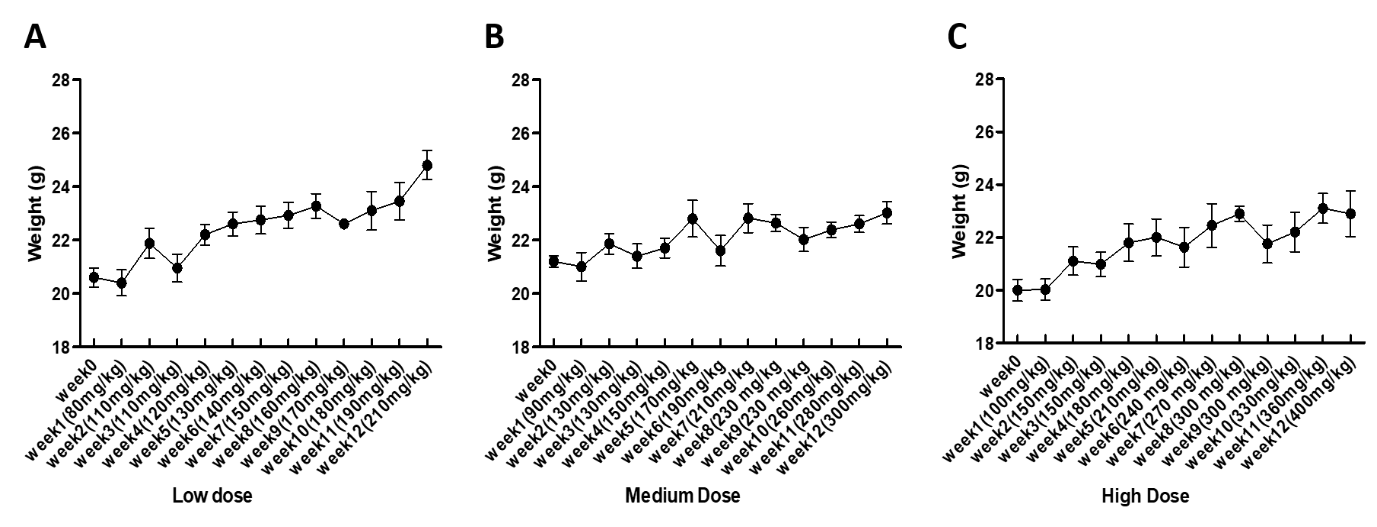


**Figure S 1.** Weight adapted TAA administration in mice at each week. A. Low Dose, B. Medium Dose and C. High Dose

The dosage of TAA was injected intraperitoneally according to the weight of the mice. The weight of mice was maintained almost 20 g at all points. The dosage of TAA in the time dependent study was similar to the medium dose group. The mice were sacrificed at different time points.

***Table S 1. Primers used for the study***

| **Primer Name** | **Sequence** | **Reference** |
| --- | --- | --- |
| **GAPDH** | **Forward- AATGGTGAAGGTCGGTGTGAAC**  **Reverse- GAAGATGGTGATGGGCTTCC** | ^1^ |
| **HBV1.2** | **Forward- CTC GTG GTG GAC TTC TCT C**  **Reverse- CTG CAG GAT GAA GAG GAA** | ^2^ |

**Table 1.** Primer target name and forward and reverse sequence of the primers used for the study

To check the HBV DNA in the transfected cells as well as in the liver at different time point, primers were used to quantify it by qPCR.

***H&E***

H&E and Masson Trichrome were performed to understand the level of fibrosis in liver. H&E analysis showed a marked increase in liver fibrosis with increased time and administration of hepato-toxins. According to time and administration of TAA/EtOH, the degeneration of hepatocytes and inflammatory infiltration was found. The bridging fibrosis after at 15 week of administration of TAA/EtOH shows a time dependent elevation of fibrosis in HBV mice compared to other groups.

The effect of different dose of TAA/EtOH in increasing the levels of fibrosis was studied using histopathological staining H&E (Fig.S2A). The dose dependent study exhibited and increased level of fibrosis according to the increased dose of TAA/EtOH. The linear fibrosis and infiltration of inflammatory cells in to the portal and central area was found to be more in the high dose treated mice groups. All the three groups such as low dose, medium dose and high dose showed an increased fibrosis level due to the effect of hepato-toxins on HBV mice. Bridging fibrosis due to the infiltration of the inflammatory cells as well as the neutrophils surrounded to the parenchyma cells were found in all the groups. The infiltration of inflammatory cells were found more in the high dose TAA-EtOH group compared to other groups which suggest the effect of dose of hepato-toxins in the acceleration of hepatic fibrosis (Fig.S2B).

**
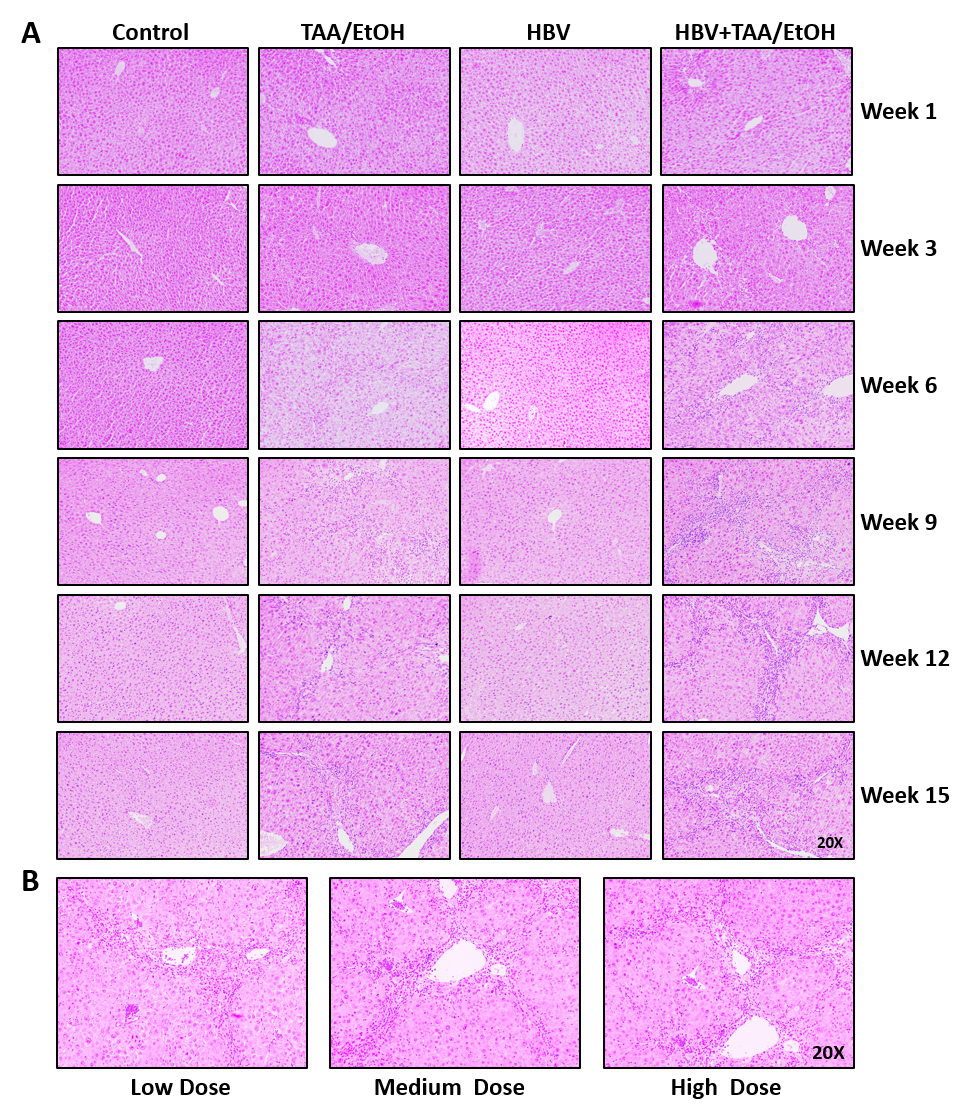
**

**Figure S 2. A. H&E image showing the bridging fibrosis in the TAA/EtOH time dependent study groups. B. H&E image showing the bridging fibrosis in the TAA/EtOH dose dependent study groups.**

***α-SMA expression***

The increased expression of α-SMA in the liver after administration of hepato- toxins such as TAA and EtOH shows the elevation in the liver fibrosis at various time points. After administration of TAA/EtOH from week 3 the α-SMA expressions were found to be increased, which suggest the effect of hepato-toxins in the elevation of liver fibrosis. The fibrosis degree was increased on the HBV/TAA group than the other groups. IHC of α-SMA showed an increased expression in the combined mice model group compared to other groups, which indicate the elevation of fibrosis in the HBV mice with hepato-toxin treatment compared to other control groups (Fig.S3.A). The α-SMA expression study was increased expression according to dosage of TAA/EtOH. The low dosage TAA/EtOH treated mice showed less expression compared to the medium dose as well as the high dose treated groups (Fig.S3.B).

**
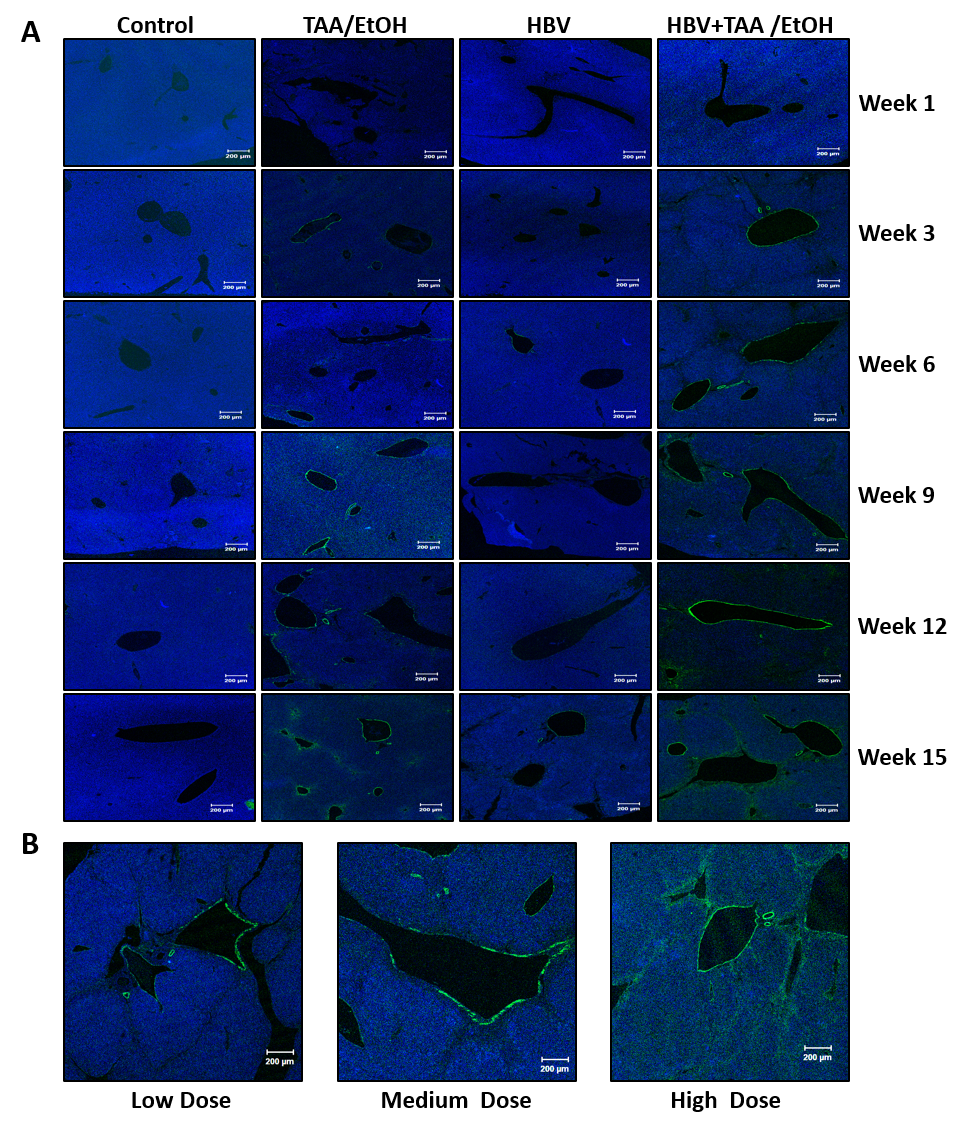
**

**Figure S3. A.** Alpha SMA Expression at different time points of TAA/EtOH administration. **B**. Alpha SMA Expression at different dosage of TAA/EtOH administration

***Masson trichrome staining analysis***

The collagen levels at different time points (Fig. S4A) of TAA and EtOH administration were confirmed using Masson trichrome staining. The bridging fibrosis was visible according to the increase in time and dosage of hepatotoxins. Linear fibrosis formation and collagen deposition were clearly visible by Masson trichrome staining of the liver. A dose-dependent study also showed an increased collagen level in all three groups. However, bridging fibrosis was more visible in the high-dose group compared to that in the low- and medium-dose groups (Fig. S4B). This indicates that the effect of HBV and dosage of hepatotoxins will increase the risk of liver fibrosis in HBV mice.

**
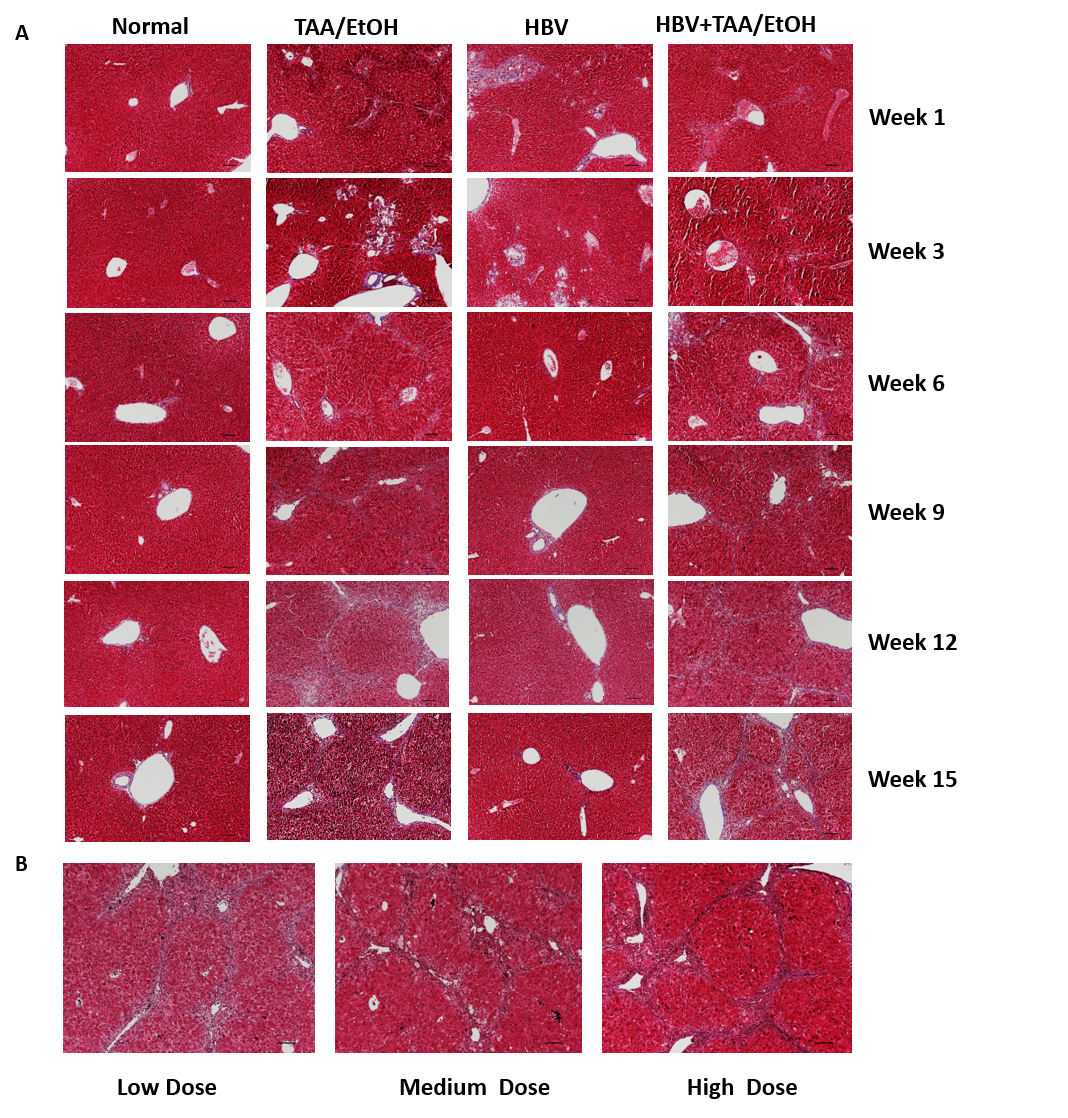
**

**Figure S4. A** Masson trichrome analysis of the time-dependent study. **B** Masson trichrome analysis of the dose-dependent study.

***Sirius Red Staining***

The collagen levels of different sample we further confirmed by Sirius red staining. The fibrosis level and the collagen deposition was analyzed for time dependent (FigS5A) as well as dose dependent (FigS5B) studies. In both the studies the bridging fibrosis was visible clearly in the combined group as the collagen was stained as red and the normal cells were stained as green. This results confirm the effect of ethanol and TAA in HBV mice. The combined mice group showed a more evident collagen deposition compared to other groups. According to time and dose the collagen deposition found to be increasing.


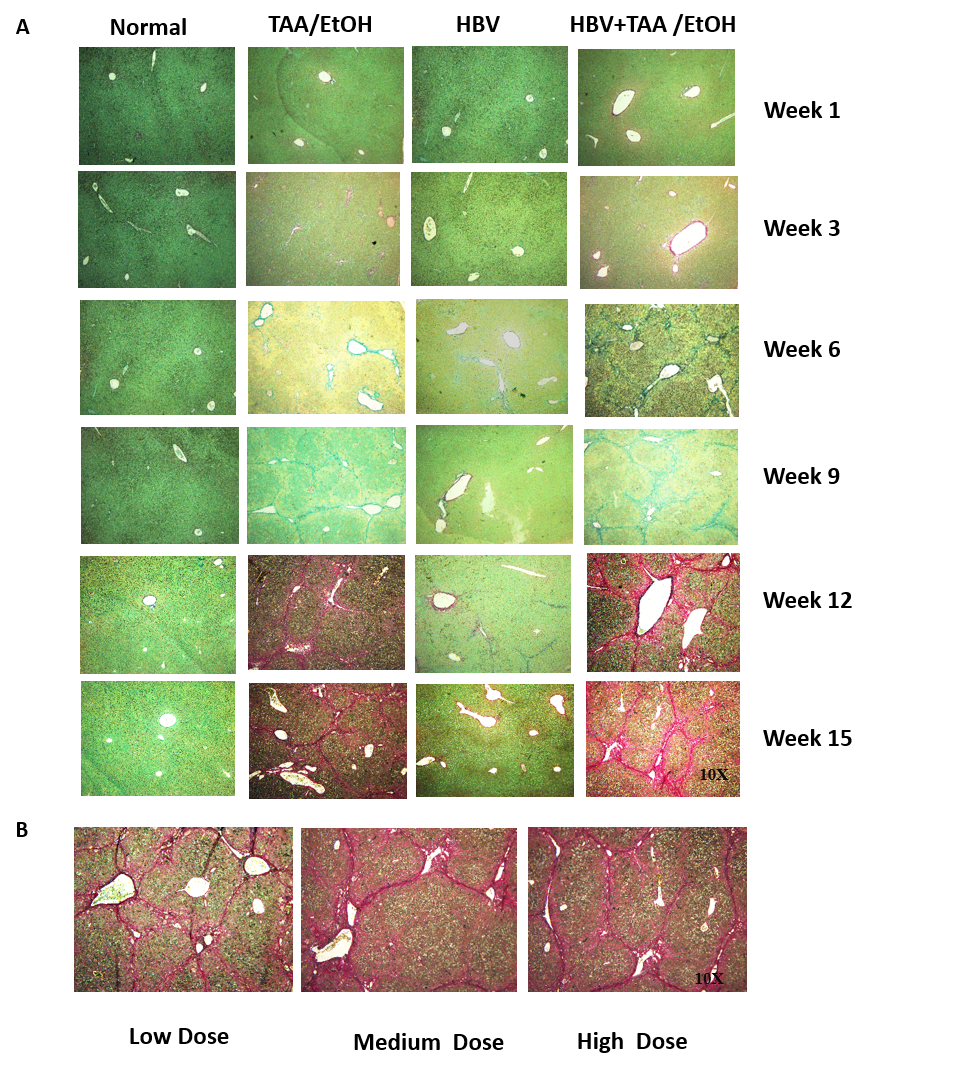


**Figure S5. A** Sirius Red staining analysis of the time-dependent study. **B** Sirius Red staining analysis of the dose-dependent study.

**Western Blotting Original Data**

**Week 1 and Week 9**

Week 1 and Week 9 α- SMA Expression


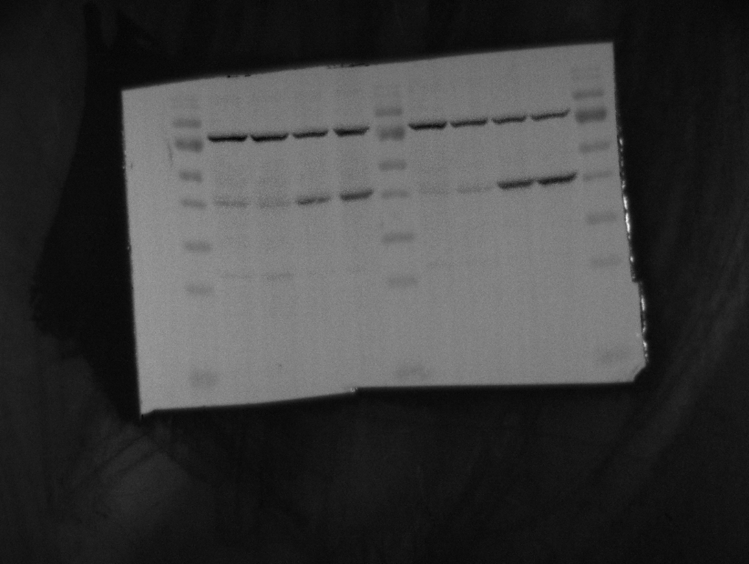


**40**

**55**

control

control

HBV

HBV

TAA/EtOH

TAA/EtOH

HBV+TAA/EtOH

HBV+TAA/EtOH

**WEEK 1**

**WEEK 9**

α- SMA

Week 1 and Week 9 β-actin Expression


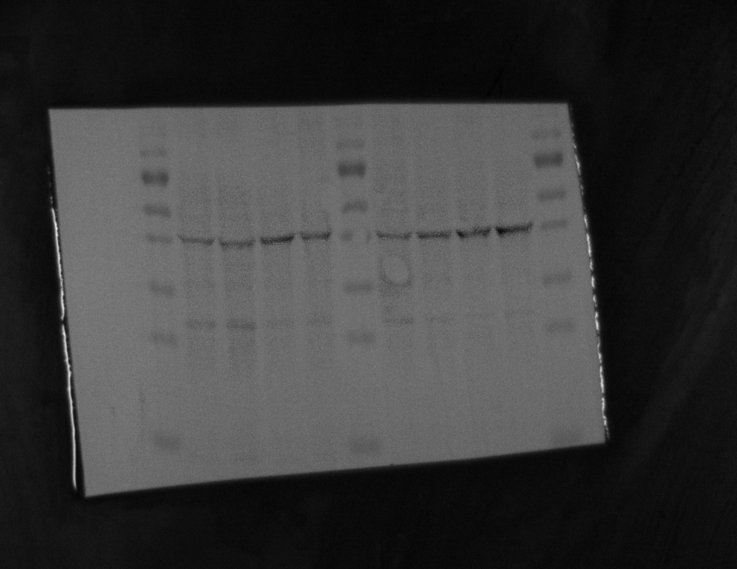


**40**

**55**

control

control

HBV

HBV

TAA/EtOH

TAA/EtOH

HBV+TAA/EtOH

HBV+TAA/EtOH

**WEEK 9**

**WEEK 1**

β- ACTIN

Week 15 α- SMA Expression


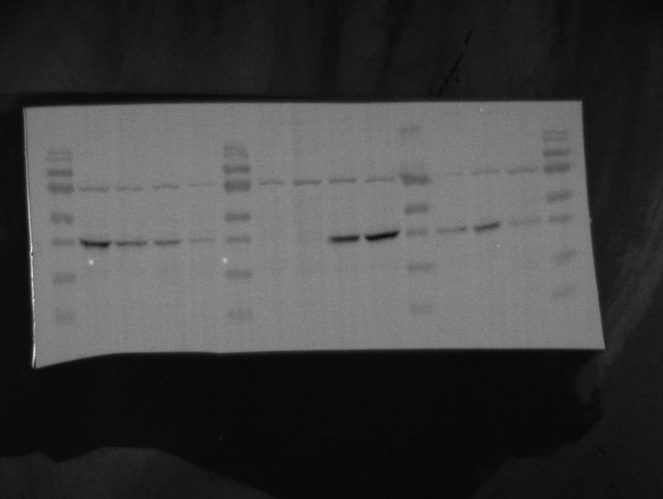


**40**

**55**

control

HBV

TAA/EtOH

HBV+TAA/EtOH

x

x

α- SMA

**WEEK 15**

Week 15β-actin Expression


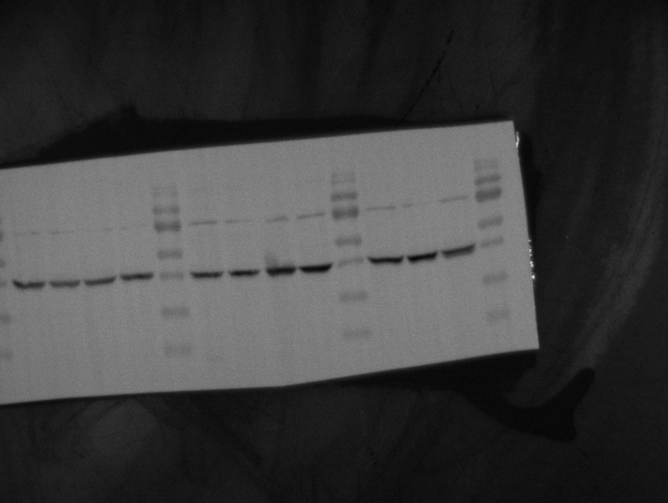


**40**

**55**

control

HBV

TAA/EtOH

HBV+TAA/EtOH

x

x

β- ACTIN

**WEEK 15**

Dosage study α- SMA Expression


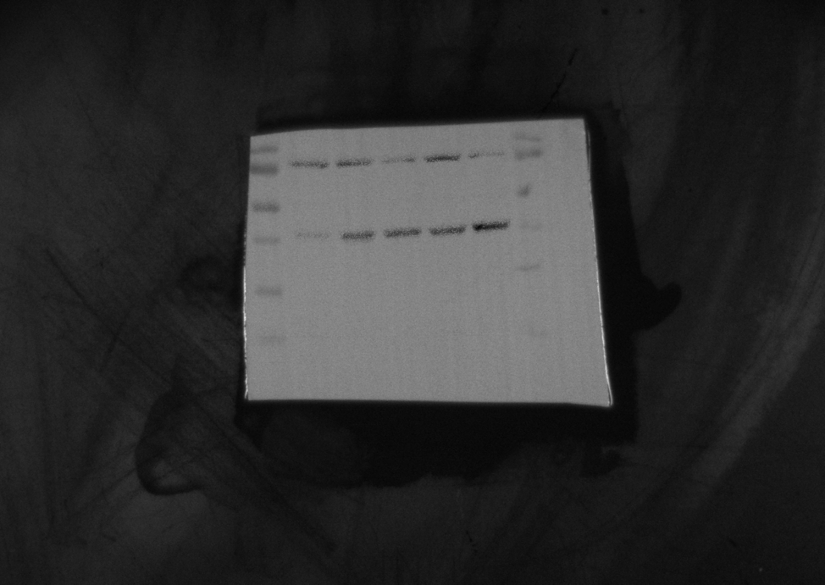


**40**

**55**

Low Dose

Medium Dose

High Dose

X

X

α- SMA

Dosage study β-actin Expression


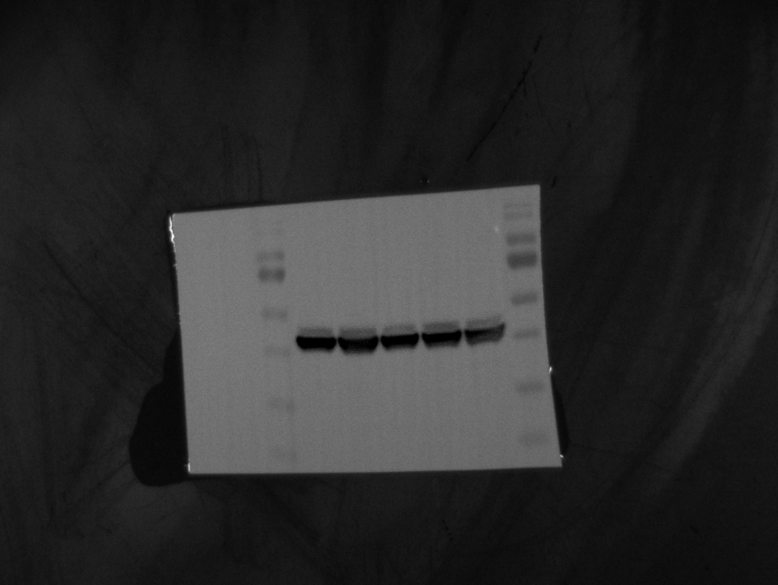


**40**

**55**

Low Dose

Medium Dose

High Dose

X

X

β- ACTIN

**References**

1. Davies. 基因的改变NIH Public Access. *Bone* **23,** 1–7 (2008).

2. Park, Y. K. *et al.* Cleaved c-FLIP mediates the antiviral effect of TNF-α against hepatitis B virus by dysregulating hepatocyte nuclear factors. *J. Hepatol.* **64,** 268–277 (2016).
